# Case Report: diffuse entire gastrointestinal tract involvement of ALK-positive anaplastic large cell lymphoma harboring JAK-STAT pathway mutations in an adolescent with leukemoid reaction

**DOI:** 10.3389/fonc.2025.1709110

**Published:** 2026-01-09

**Authors:** Sizhe Yang, Yang Dai, Qiyuan Li, Sha Zhao, Xueqin Deng, Feifan Chen, Yaping Zhang, Yufang Wang, Wenyan Zhang

**Affiliations:** 1 Department of Pathology, West China Hospital of Sichuan University, Chengdu, Sichuan, China; 2 Department of Hematology, West China Hospital of Sichuan University, Chengdu, Sichuan, China; 3 Department of Gastroenterology, West China Hospital of Sichuan University, Chengdu, Sichuan, China

**Keywords:** adolescent, anaplastic lymphoma kinase positive anaplastic large cell lymphoma, gastrointestinal tract, JAK-STAT pathway, leukemoid reaction

## Abstract

Anaplastic lymphoma kinase-positive anaplastic large cell lymphoma (ALK+ ALCL) is an aggressive mature T-cell non-Hodgkin lymphoma. Its typical characteristics include positive CD30 and ALK expression detected by immunohistochemistry, and it is often accompanied by ALK gene translocation, among which the chromosomal translocation t (2;5) is the most common. This disease predominantly affects young individuals, with extranodal involvement being relatively common. However, cases involving the digestive tract are relatively rare. We report a rare case of a 14-year-old female with ALK+ALCL presenting with fever and peripheral blood leukocytosis as initial manifestations and complicated by diffuse involvement of the entire gastrointestinal tract. The patient showed significant remission having completed 4 chemotherapy sessions. In further studies, RNA sequencing confirmed the presence of *NPM1::ALK* gene fusion in this case; whole-exome sequencing (WES) detected somatic mutations in multiple genes of the JAK-STAT pathway (including *JAK1, PTPN6, MTOR*, and *TYK2*). It is noteworthy that such genetic alterations are more commonly observed in ALK-negative anaplastic large cell lymphoma (ALK-ALCL) but are less commonly reported in ALK+ALCL.

## Introduction

Anaplastic large cell lymphoma (ALCL) is a rare type of Non-Hodgkin lymphoma (NHL), accounting for 1% - 3% of NHLs and approximately 15% of T-cell lymphomas (TCL) (1, 2). ALCL is categorized into three subtypes: ALK-positive anaplastic large cell lymphoma (ALK+ALCL), ALK-negative anaplastic large cell lymphoma (ALK-ALCL), and breast implant-associated anaplastic large cell lymphoma (BIA-ALCL) (3).

ALK+ALCL is characterized by the positive expression of ALK protein and ALK gene translocation (3). Among them, systemic ALK+ALCL is more prevalent in young populations, with a higher proportion of male patients (1).

Approximately 80% of patients with ALK+ALCL harbor the chromosomal translocation t(2;5), which gives rise to the *NPM1::ALK* fusion gene (4). The formation of this fusion gene triggers the autophosphorylation of ALK protein, thereby activating multiple signaling pathways, such as those mediated by STAT3, PLCγ, and MEK (5).

Extranodal involvement is extremely common in patients with ALK+ALCL with the involvement rates of 26% for the skin, 14% for the bones, 15% for the soft tissues, 12% for the lungs, and 8% for the liver (2, 6–8).

Previous reports indicate that TCL of the digestive tract is a relatively uncommon entity, constituting 4.5% (38/836) of all digestive tract lymphomas. Notably, ALK+ALCL only represents a mere 0.12% (1/836) of digestive tract lymphomas (9). Primary gastrointestinal T/NK cell lymphoma (GI-TNKL) with multiple sites is not uncommon, accounting for approximately 31.6% (12/38) (9).

Leukemoid reaction (LR) is defined as a significant increase in peripheral blood leukocytes (exceeding 40×10^9^/L), accompanied by marked “left shift” (10). It is commonly seen in severe infections, malignant tumors, acute trauma or burns, acute hemolysis, drug reactions, etc (11). The diagnosis of paraneoplastic leukemoid reaction(PLR) posed considerable challenges, given the potential association of infection and the use of corticosteroids/growth factors in such patients (12).

Herein, one challenging case of ALK+ALCL is reported. The patient’s initial presentation was LR, with a life-threatening condition and concurrent extensive erythematous papules on the skin. Endoscopic findings revealed diffuse mucosal lesions involving the entire gastrointestinal tract. Relevant reports have been reported to be extremely scarce so far.

## Case presentation

A 14-year-old girl presented with a one-month history of fever, paroxysmal abdominal pain, vomiting, and diarrhea. The fever is intermittent, with the highest body temperature reaching 39°C. Physical examination revealed a symmetrical eruption of diffuse erythematous maculopapules, 0.1–0.5 cm in diameter, slightly raised and non-blanching, most prominent on the lower limbs (**Supplementary File S3**). The remainder of the examination was unremarkable. Laboratory tests revealed white blood cell 59.18× 10^9^/L (normal range:3.5-9.5×10^9^/L), neutrophilia 95.5% (40-75%), platelet 599× 10^9^/L (150-407), haemoglobin 127g/L. Coagulation tests showed prothrombin time of 38.5 seconds (9.6-12.8), activated partial thromboplastin time of 39.0 seconds. Inflammatory markers were detected as C-reactive protein 108.00 mg/L(0-6), procalcitonin 0.711ng/mL (<0.046), interleukin-6 43.90 pg/mL(0-7), and soluble interleukin-2 receptor 166600U/mL (223-710).

Considering the fever and remarkably inflammatory markers, infections related tests were conducted which included blood culture, blood and bone marrow next-generation sequencing (NGS), routine stool test+ occult blood+ parasites, sputum culture, fungal G/GM test, CMV, EBV, tuberculosis related tests. However, no positive results were obtained.

Moreover, chest and abdominal CT scans were performed which revealed normal image in chest, while abdominal scan suggested swelling and edema of multiple segments of the small intestine and colon, peritoneal effusion, peritonitis, and pelvic effusion.

**Figure 1:**
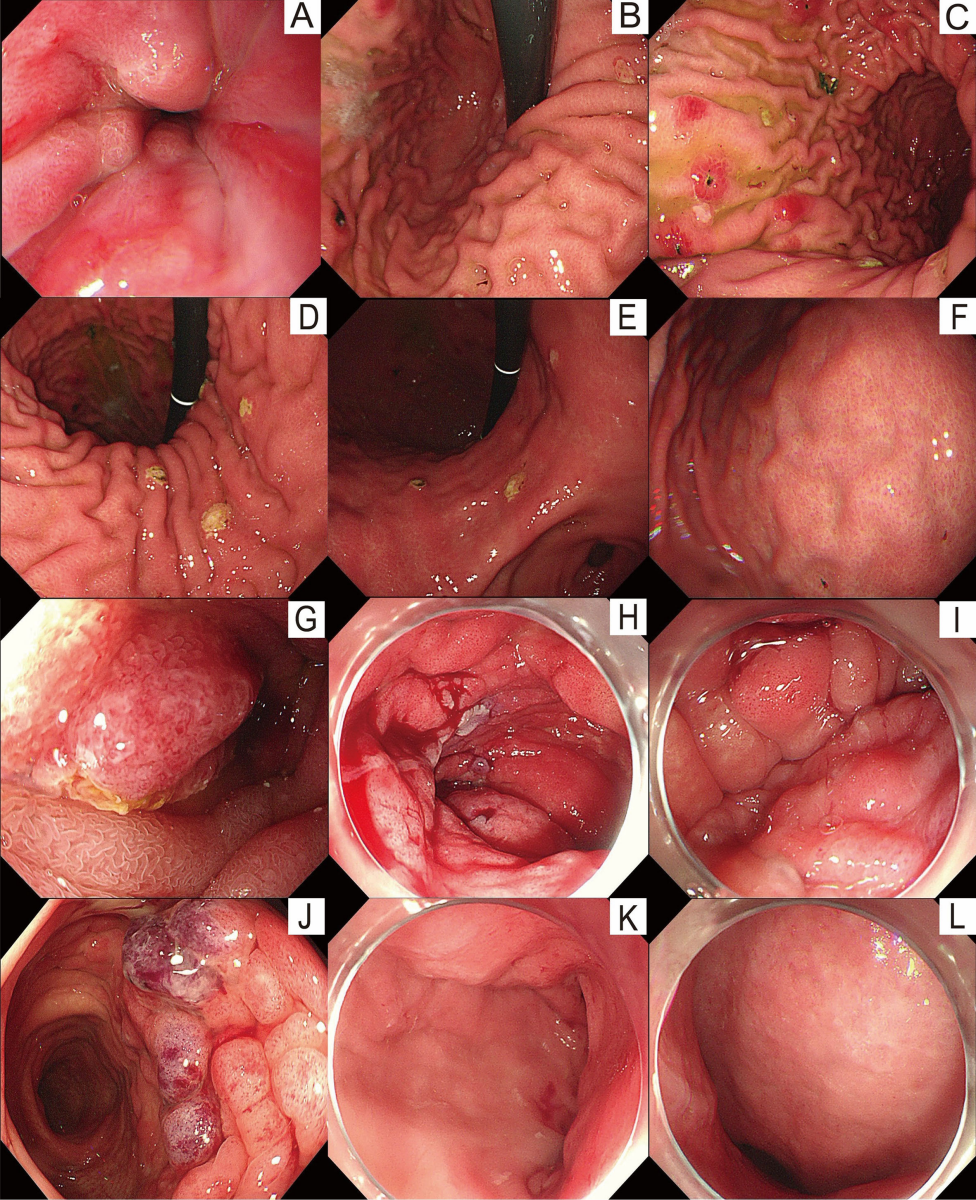
Pre-treatment endoscopic findings. **(A)** Distal esophagus: multiple linear areas of erythema and congestion. **(B)** Gastric fundus: smooth mucosa with a greenish mucus lake. **(C)** Gastric body: thickening of the greater-curvature folds with edematous mucosa and scattered round erosions (0.3–0.6 cm) with associated erythema. **(D)** Gastric angle: preserved morphology with smooth mucosa. **(E)** Gastric antrum: markedly edematous mucosa with scattered erosive foci. **(F)** Duodenal bulb: mildly edematous mucosa without ulceration. **(G)** Descending duodenum: swollen mucosa with an enlarged, rough, and hyperemic major papilla. **(H)** Ascending colon: nodular mucosal elevations with erythematous swelling, and luminal narrowing prevented further endoscope passage. **(I)** 60 centimeters from the anal verge: patchy, rough, and thickened mucosa with segmental involvement and granular erythematous changes, and the lumen was mildly narrowed. **(J)** Descending colon: patchy mucosal thickening and roughness with segmental distribution and granular erythema, with slight luminal narrowing. **(K)** Sigmoid colon: mildly swollen mucosa with preserved vascular pattern, regular haustra, a patent lumen, and no ulceration or neoplasia. **(L)** Rectum: mildly edematous mucosa with an intact vascular pattern, regular haustration, and no luminal narrowing, ulceration, or mass lesion.

Endoscopic findings revealed mucosal congestion in the lower esophagus, swelling of the gastric body and antrum with scattered 0.3-0.6 cm erosions, mucosal swelling in the duodenal bulb and descending part, along with an enlarged duodenal papilla with rough mucosa. Additionally, generalized swelling was observed throughout the colon. Ascending colon presented nodular, erythematous elevations accompanied by swelling and congestion with a relatively smooth overlying mucosa. The lumen was too narrowed to allow continuous observation during colonoscopy. At 60 cm from the anal verge (mid-transverse colon), in the sigmoid colon, and at the sigmoid–descending junction, the mucosa showed patchy, segmental areas of coarseness and swelling. The affected mucosa had a granular appearance, was erythematous, bled easily on contact, and exhibited superficial erosions; mild luminal narrowing was also noted (**Figure 1**).

**Figure 2:**
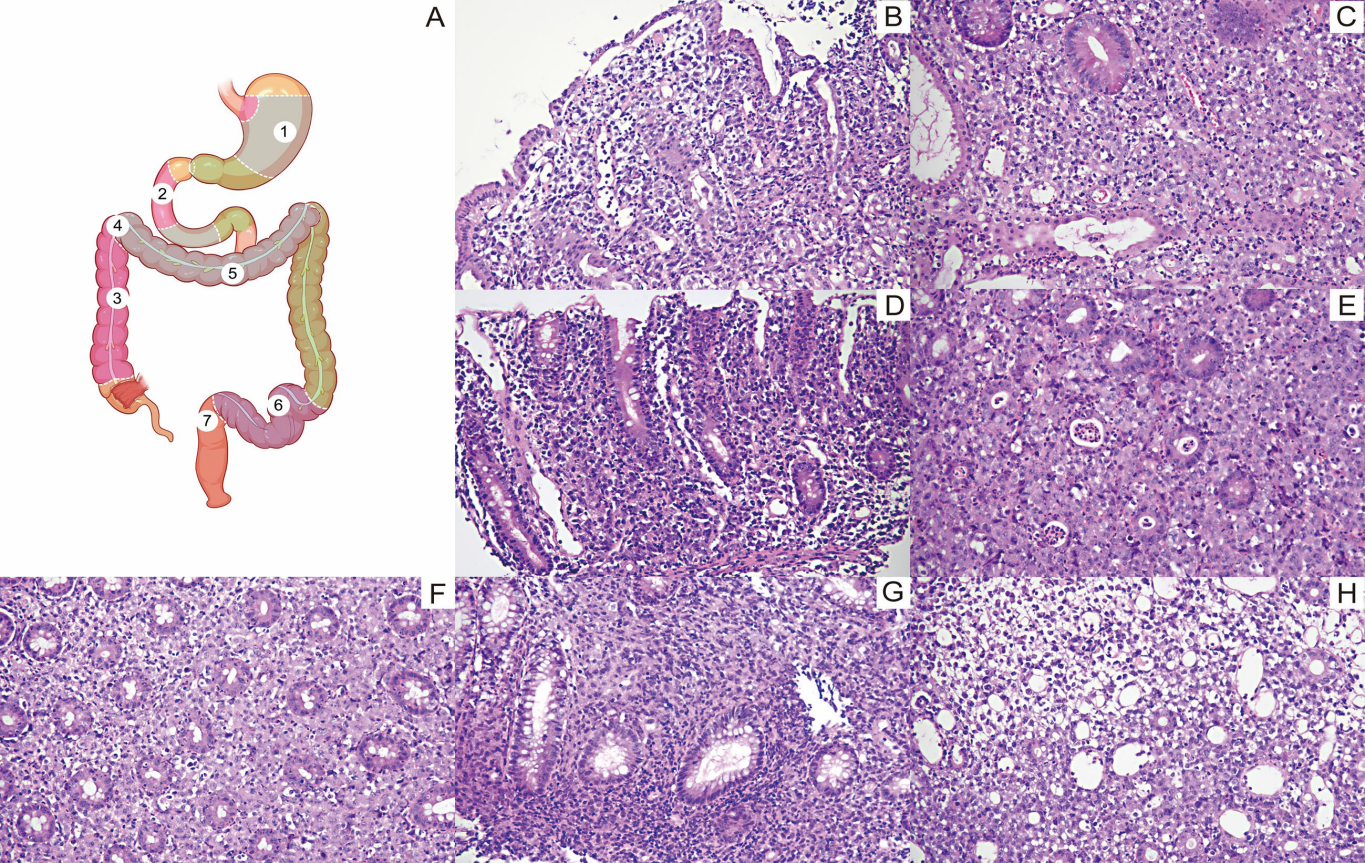
Endoscopic biopsy sites and histologic findings. **(A)** Schematic illustration of endoscopic sampling sites, including the gastric body, duodenal papilla, ascending colon, 60 cm from the anal verge, transverse colon, sigmoid colon, and the rectosigmoid junction, all biopsies revealed tumor involvement. **(B)** Gastric body biopsy (H&E, 200×): mucosal erosion with infiltration of large, cytoplasm-rich lymphoid cells between gastric glands, showing marked atypia and abnormal nuclear-to-cytoplasmic ratios. **(C)** Duodenal papilla biopsy (H&E, 200×): dense infiltration of atypical lymphoid cells with an acute inflammatory background. **(D)** Ascending colon biopsy (H&E, 200×): infiltration of atypical lymphoid cells extending from the lamina propria into the muscularis mucosae. **(E)** Biopsy at 60 cm from the anal verge (H&E, 200×): abundant cytoplasm-rich lymphoid cells infiltrating between glands, with an acute inflammatory background and crypt abscess formation. **(F)** Transverse colon biopsy (H&E, 200×): lymphoid-cell infiltration with acute inflammation. **(G)** Sigmoid colon biopsy (H&E, 200×): markedly reduced colonic glands with interglandular infiltration by tumor cells/lymphoid cells. **(H)** Rectosigmoid junction biopsy (H&E, 200×): mucosal erosion with lymphoid-cell infiltration.

**Figure 3:**
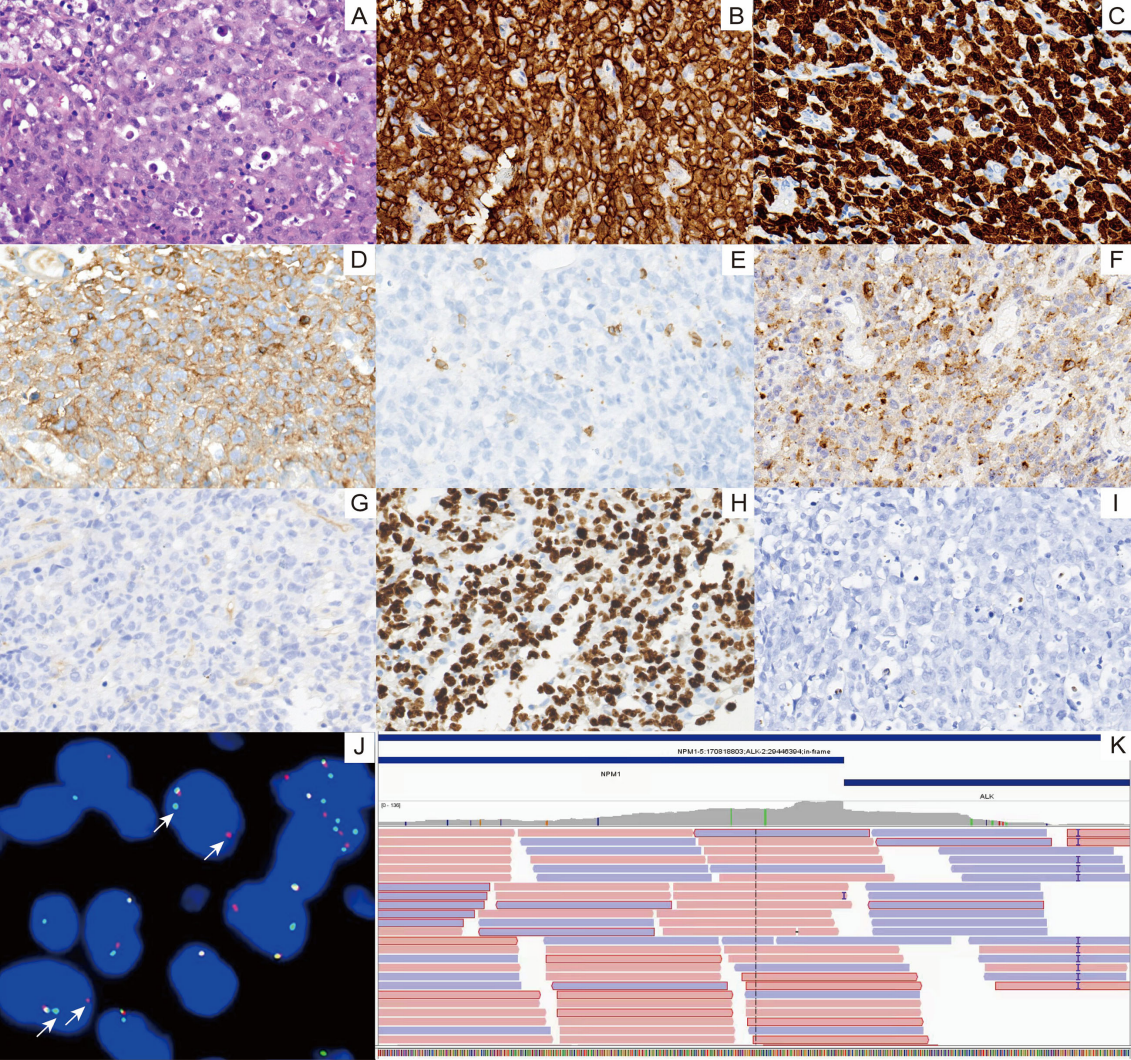
Pathological findings of the tumor. **(A)** The tumor cells are large in size with abundant cytoplasm, and their nuclei are large and horseshoe-shaped, with prominent and multiple nucleoli (H&E, 400×). **(B)** Tumor cells exhibited CD30 positivity on the cell membrane and in the cytoplasm (IHC, 400×). **(C)** Strong ALK-1 positivity in the nucleus and cytoplasm of tumor cells (IHC, 400×). **(D)** Tumor cells are CD4 positive (IHC, 400×). **(E)** Tumor cells are CD8 negative (IHC, 400×). **(F)** Tumor cells showed granular positivity for Granzyme B in the cytoplasm (IHC, 400×). **(G)** Tumor cells are negative for TDT (IHC, 400×). **(H)** Ki-67 labeling index of tumor cells is approximately 80% (IHC, 400×). **(I)** EBER *in situ* hybridization is negative (400×). **(J)** FISH analysis demonstrates ALK gene rearrangement with clear separation of red and green signals. **(K)** RNA-seq detects an *NPM1::ALK* fusion gene formed by the recombination of exon 4 of *NPM1* (ENST00000296930.5/NM_002520.6, chr5:170818803: +) and exon 20 of *ALK* (ENST00000389048.3/NM_004304.4, chr2:29446394: -).

Seven biopsies were performed throughout the entire gastrointestinal tract. Atypical lymphocytes infiltrate between the intrinsic glands (**Figure 2**). Immunohistochemistry (IHC, Envision) of tumor cells showed: CK (-), CD20(-), CD3(-), CD4(+), CD8(-), EMA(+),CD43(+), CD30(+), TIA-1(+), Granzyme B (+), TDT (-), ALK-1(+), CD5(+), Ki-67(80%). Epstein-Barr Virus Encoded RNA (EBER) *in situ* hybridization (ISH) was negative. Clonal amplification peaks were detected within the target bands by TCRG gene rearrangement testing. *ALK* rearrangement was evaluated using a dual-color break-apart probe (GSP *ALK* [Centromere]/GSP *ALK* [Telomere], probe code F.01552-01; LBP SYSTEM, China). ALK-FISH was positive (+), and RNA sequencing revealed the *NPM1::ALK* fusion gene (**Figure 3**) (Watchmaker RNA Library Preparation Kit to extract RNA and Illumina NovaSeq X Plus platform to sequence). ALK+ALCL was diagnosed. While bone marrow biopsy was negative.

WES revealed somatic mutations in the *JAK1*, *PTPN6*, *MTOR*, and *TYK2* genes in this patient. (**Supplementary File S1**) (Agilent SureSelect XT Capture Kit for library preparation and sequenced on an Illumina platform). The methods for FISH, RNA-seq, and WES are presented in **Supplementary File S2**.

Due to a high tumor burden, fractionated E-CHP chemotherapy was initiated. The specific regimen was etoposide 80 mg on day 1, cyclophosphamide 600 mg on day 2 and 400 mg on day 4, liposomal doxorubicin 20 mg on days 3 and 5, and methylprednisolone 40 mg daily on days 1–10. Vincristine was deliberately omitted to avoid neurotoxicity that might worsen the patient’s abdominal distension. Chidamide 20 mg twice weekly was added.

Fever resolved, abdominal pain and distension improved markedly, and ventilatory support was weaned. Skin lesions also regressed noticeably (**Supplementary File S3**). Three weeks later, CNS prophylaxis was given via lumbar puncture with intrathecal cytarabine, methotrexate, and dexamethasone, followed by course 2 of chidamide-CHOP. That’s chidamide 20 mg twice weekly, cyclophosphamide 1,100 mg on day 1, liposomal doxorubicin 50 mg on day 1, vindesine 4 mg on day 1, and prednisone 50 mg twice daily on days 1–5. Courses 3 and 4 of identical chidamide-CHOP were delivered at 3-week intervals. Considering the milder neurotoxicity, vindesine rather than vincristine was chosen.

**Figure 4:**
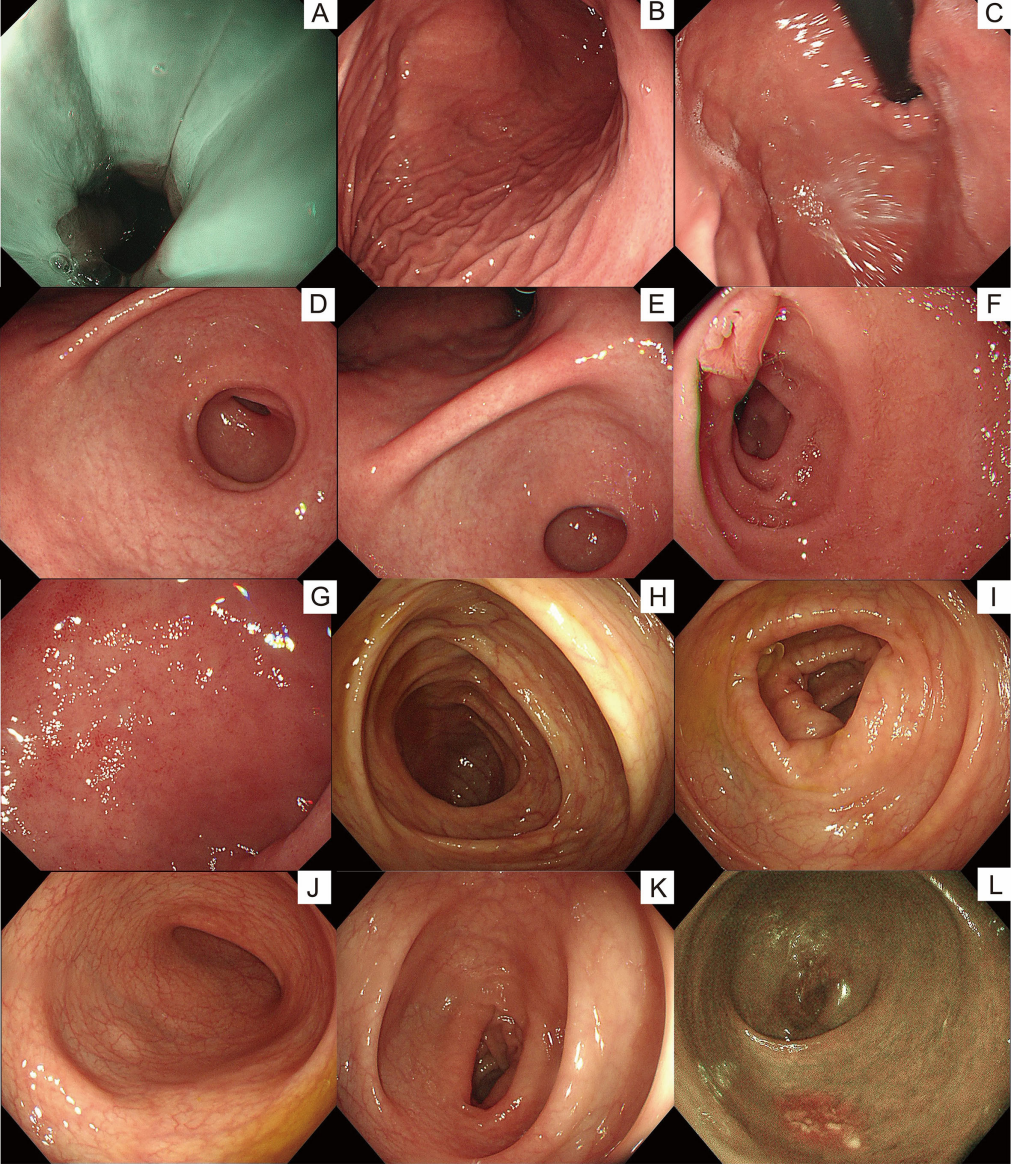
Endoscopic findings after 4 courses of chemotherapy. **(A)** Distal esophagus: smooth, moist mucosa with clear vasculature. **(B)** Gastric fundus: smooth mucosa with a clear mucus lake. **(C)** Gastric body: gyrus-like greater curvature rugae with orange-red mucosa. **(D)** Gastric angle: intact morphology and smooth mucosa. **(E)** Gastric antrum: normal peristalsis, mucosa with red-white alternation, macular changes, and visible submucosal vessels, with soft biopsy texture. **(F)** Duodenal bulb: smooth mucosa with no ulcers detected. **(G)** Descending duodenum: smooth mucosa with no ulcers or neoplasms identified. **(H)** Ascending colon: smooth and clean mucosa with uniform orange-red hue, clear and naturally distributed vasculature, regular haustra, patent lumen with no stenosis, and no ulcers, erosion, or neoplasms. **(I)** 60 cm from anus: smooth mucosa with normal luster and uniform orange-red pigmentation, clear vascular network, regular symmetrical haustra, patent lumen with no stenosis, and no ulcers, polyps, or neoplasms. **(J)** Descending colon: normal smooth mucosa with bright orange-red hue, clear vasculature, intact regular haustra, spacious lumen with no stenosis, and no ulcers, polyps, or neoplasms. **(K)** Sigmoid colon: smooth and flat mucosa with good luster and uniform orange-red pigmentation, clear natural vasculature, regular haustra, unobstructed lumen with no stenosis, and no ulcers, polyps, or neoplasms. **(L)** Rectum: smooth and clean mucosa with uniform orange-red hue, clear distinguishable vasculature, regular haustra, patent lumen with no stenosis, and no ulcers, polyps, or neoplasms.

After four courses, interim restaging showed complete metabolic remission. Whole-body Positron Emission Tomography/Computed Tomography (PET/CT) was negative (Deauville 1), and gastroscopy plus colonoscopy were normal (**Figure 4**). Four additional courses of the same regimen consolidated the response. The patient remains in complete remission. During follow-up, the patient reported feeling well without any discomfort and was able to carry out normal academic and daily activities. She is now receiving chidamide 20 mg twice weekly as maintenance therapy, planned for 2 years.

## Discussion

ALK+ ALCL occurring in the gastrointestinal tract is a very rare entity (13). We retrospectively reviewed ALCL cases diagnosed in the Department of Pathology, West China Hospital, Sichuan University from November 21, 2008 to May 23, 2025. A total of 165 ALCL cases were identified, including 70 ALK-ALCL and 95 ALK+ALCL cases. Gastrointestinal involvement was observed in 6 patients (1 ALK-ALCL and 5 ALK+ALCL). During the same period, 2,568 gastrointestinal lymphomas were diagnosed, with ALK+ ALCL representing 0.19% of these cases.

It is reported that GI-TNKL mostly presents as masses (about 47.6%), deep (about 23.8%) or superficial ulcers (23.8%) under endoscopy, and only a few cases show almost normal manifestations (9). Gastrointestinal mantle cell lymphoma (MCL) typically presents as multiple mucosal polyps involving multiple intestinal segments, most commonly in the small intestine, and was formerly referred to as multiple lymphomatous polyposis (LP) (14, 15). These polypoid lesions can be multifocal or diffuse and have also been described in follicular lymphoma (FL) and mucosa-associated lymphoid tissue (MALT) lymphoma (14), and these three subtypes of lymphoma rarely occur in children (3). However, the endoscopic manifestations of gastrointestinal lymphomas involving the entire digestive tract have been rarely reported. Zhang et al. and Cheng et al. have reported similar cases of mantle cell lymphoma featuring diffuse nodular or polypoid lesions in the entire gastrointestinal tract in middle-aged and elderly males (16, 17).

In contrast, in our case, gastroscopy and colonoscopy showed swelling and redness of the entire mucosa, with multiple protuberances. The protuberances and roughness of the intestinal mucosa were distributed in a segmental pattern. Given the patient’s clinical manifestations of fever and skin rashes, as well as the blood picture showing a leukemoid reaction, it is difficult to distinguish between connective tissue diseases such as allergic purpura and vasculitis, severe infections, and tumors. Endoscopic biopsy is particularly important for the diagnosis of this case. Meanwhile, it is crucial to perform multi-site biopsies via endoscopy, as the pathological results of multiple mucosal lesions in the intestine may indicate different types of tumors (18).

Studies have shown that when the cause of leukemoid reaction is tumor, the mortality rate is extremely high, approximately 76% (12).In most cases, the occurrence of PLR is often associated with cytokine-secreting tumors (CST) and tumor microenvironment (TME) (12). The most commonly secreted cytokine is granulocyte colony-stimulating factor (G-CSF). In addition, GM-CSF, IL-1a, IL-1b, IL-3, IL-6 and tumor necrosis factor(TNF)-α have also been reported (19). Sueki et al. summarized 12 cases of ALCL with leukemoid reaction. Among them, 5 cases showed elevated G-CSF in serum cytokine tests. 8 cases underwent immunohistochemical staining of tumors, with 5 cases positive for TNF-α and 7 cases positive for G-CSF. The prognosis of such cases is extremely poor, with only 1 case achieving complete remission and the rest dying within 6 months (20). Although we were unable to detect the level of G-CSF in this patient, we found that the levels of IL-6 and IL-2 receptor in their serum were significantly elevated.

Anomalous changes in cytokine profiles occur in ALCL (21). Studies have revealed that the concentrations of sIL-2R, IL-10, and IL-17a in patients with ALCL at the time of diagnosis are significantly higher than those in patients with B-NHL and ALCL patients in remission (21). Furthermore, the IL-6 concentration is significantly higher in ALCL patients with B symptoms or elevated CRP, suggesting that IL-6 is involved in the inflammatory process of the disease (21, 22).

Tumor and plasma samples from the patients were sent for WES. Somatic mutations in multiple genes of the Janus kinase (JAK) signal transducer and activator of transcription (JAK-STAT) pathway (*JAK1, PTPN6, MTOR, TYK2*) were identified. Notably, the sustained activation of the JAK-STAT pathway is one of the core pathogenesis mechanisms in both ALK+ALCL and ALK-ALCL. For ALK+ALCL, ALK fusion genes trigger the autophosphorylation of ALK proteins, which in turn activates STAT3 (2, 23). A key mechanism of action of STAT3 is to promote the proliferation and survival of tumor cells by mimicking physiological growth-promoting signals (IL-2 and T-cell receptor (TCR) signaling pathways), thereby playing a critical role in ALCL formation (24). For ALK-ALCL, it has been reported that approximately 20% of cases harbor mutations in *JAK1* and *STAT3* genes, and about half of the cases exhibit STAT3 activation (23). Shaw T. I. et al. performed NGS on 46 pediatric cases of ALK+ALCL, and no genomic alterations in *JAK1* or *STAT3* were detected (25).

The activation of the JAK-STAT pathway plays a complex yet crucial role in the differentiation and development of immune cells, as well as in the maintenance of immune system homeostasis, while also influencing disease progression (26). Ruxolitinib targets the JAK-STAT signaling pathway and can simultaneously inhibit two key pathways: one is the downstream pathway that mediates colony-stimulating factors to promote neutrophil proliferation, and the other is the upstream pathway that induces STAT-3-mediated IL-6 synthesis (27). Based on the above-mentioned mechanism of action, this drug can be used for the treatment of paraneoplastic leukemoid reactions (28).

Based on this, we speculate that the occurrence of leukemoid reaction in this case of ALK+ALCL may be associated with the complex inflammatory response caused by the active state of its cytokines (29). In addition, for the phenomenon of significantly elevated white blood cells in the patient reported in this study, we speculate that it may also be related to gene mutations in the JAK-STAT pathway.

## Conclusion

We report a case of ALK+ALCL presenting with fever, intestinal obstruction as the main symptoms, and the results of the blood routine test indicate a leukemoid reaction. The entire gastrointestinal tract being affected, and significantly distinctive and rare endoscopic findings being identified. The presence of the *NPM1::ALK* gene fusion was revealed in this patient by RNAseq. Meanwhile, multiple somatic mutations in the JAK-STAT pathway were shown by WES results to be carried by the patient, which have not been clearly reported in ALK+ALCL. We speculate that the leukemoid reaction in this patient may be associated with the abnormal activation of ALCL cytokines and mutations in the JAK-STAT pathway.

## Data Availability

The original contributions presented in the study are included in the article/**Supplementary Material**. Further inquiries can be directed to the corresponding authors.
