# Supplementary material for: Case Report: diffuse entire gastrointestinal tract involvement of ALK-positive anaplastic large cell lymphoma harboring JAK-STAT pathway mutations in an adolescent with leukemoid reaction
Source: Front Oncol. 2026 Jan 9;15:1709110. doi: 10.3389/fonc.2025.1709110 (PMC12827520; doi:10.3389/fonc.2025.1709110)
Supplement: Supplementary file 1 [file DataSheet1.docx]

**Methods for ALK-FISH, RNA-seq, and WES**

**ALK fluorescence in situ hybridization**

ALK rearrangement was evaluated using a dual-color break-apart probe (GSP ALK [Centromere]/GSP ALK [Telomere], probe code F.01552-01; LBP SYSTEM, China) by fluorescence in situ hybridization (FISH), as previously described.The probe targets the ALK gene locus at chromosome 2p23, with the centromeric (5′) and telomeric (3′) probes hybridizing to regions flanking the ALK breakpoint. Fifty non-overlapping tumor cell nuclei were examined for each case. ALK gene rearrangement was indicated by a split signal, defined as a separation of the red and green signals by at least two signal diameters. A case was considered positive when more than 15% of the assessed nuclei showed split signals.

**RNA sequence**

RNA was extracted from Formalin-Fixed, Paraffin-Embedded (FFPE) tumor samples containing more than 20% tumor cells. Ribosomal RNA and globin RNA were efficiently depleted from total RNA using sequence-specific probes to enrich mRNA. Because FFPE fixation can cause RNA fragmentation and damage, the enriched mRNA was first repaired and then thermally fragmented into appropriate sizes. First- and second-strand cDNA synthesis was subsequently performed, followed by ligation of sequencing adaptors to both ends of the cDNA fragments. The adaptor-ligated products were purified and amplified to generate whole-transcriptome libraries suitable for high-throughput sequencing, using the Watchmaker RNA Library Preparation Kit.

The libraries were sequenced on an Illumina NovaSeq X Plus platform with 150-bp paired-end reads. VarDict was used to identify candidate SNVs and indels, and high-confidence variants shared across multiple analytic steps were selected for subsequent annotation. STAR was used for alignment and fusion gene assessment, while STAR-Fusion and Arriba were applied for fusion gene detection.

**Whole Exome Sequencing**

In this study, WES was employed to analyze somatic mutations, with sample types including fresh plasma and FFPE tumor tissues. After DNA extraction, libraries were constructed using the Agilent SureSelect XT Capture Kit: genomic DNA was fragmented into 150-220 bp via the Covaris S220 system, followed by end repair, 3' adenylation, adapter ligation, and PCR enrichment. The resulting products were hybridized with the SureSelect Capture Library, and target exonic regions were captured using streptavidin-modified magnetic beads. The captured products were subjected to PCR amplification, and their quality and concentration were verified using the Bioanalyzer 2100 and Qubit Fluorometer. Raw sequencing reads were filtered by the fastp software to remove adapter sequences, low-quality reads, and those containing ≥5 N bases, yielding clean reads. The clean reads were aligned to the human reference genome (hg19/hg38) using the BWA software, followed by format conversion with SAMtools and PCR duplicate removal with Picard. Base quality recalibration and SNP/InDel detection were performed using GATK with a filtering threshold of QD≥2.0. For somatic mutation detection, MuTect was used to identify somatic SNVs and InDels. All variant sites were annotated to databases including Refseq, COSMIC, and gnomAD using the Annovar software. Germline mutations were filtered out by comparing tumor tissues with fresh plasma (normal control) samples, ultimately obtaining the tumor-specific somatic mutation profile.
