# Supplementary material for: Case Report: diffuse entire gastrointestinal tract involvement of ALK-positive anaplastic large cell lymphoma harboring JAK-STAT pathway mutations in an adolescent with leukemoid reaction
Source: Front Oncol. 2026 Jan 9;15:1709110. doi: 10.3389/fonc.2025.1709110 (PMC12827520; doi:10.3389/fonc.2025.1709110)
Supplement: Supplementary file 2 [file DataSheet2.docx]

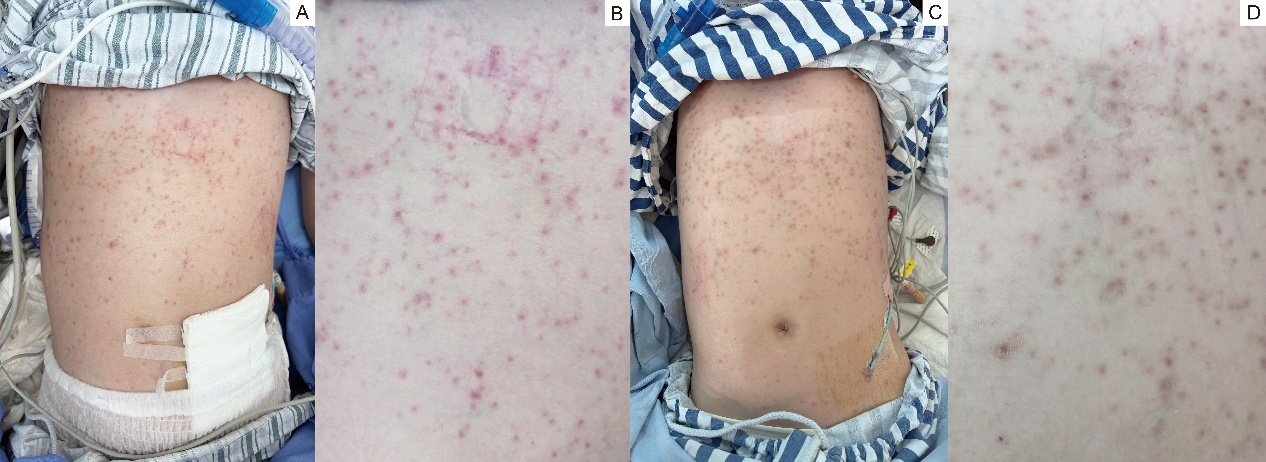


Supplementary Figure1. Cutaneous observations before and after treatment. (A) Before treatment, non-blanchable, slightly elevated erythematous cutaneous lesions (0.1–0.5 cm in diameter) were observed on the extremities and trunk. (B) Before treatment, the lesions exhibited ill-defined borders, a smooth texture without exudation or necrosis, and were asymptomatic (no pruritus, tenderness, or burning sensation). (C) Four days after treatment, the lesions were markedly darkened in color, flattened, and reduced in size with diminished infiltration; no new lesions developed. (D) The lesions became flattened, reduced in size, darkened in color, with the emergence of a small amount of dry, thin, and easily removable scales.
