# Supplementary material for: Case Report: diffuse entire gastrointestinal tract involvement of ALK-positive anaplastic large cell lymphoma harboring JAK-STAT pathway mutations in an adolescent with leukemoid reaction
Source: Front Oncol. 2026 Jan 9;15:1709110. doi: 10.3389/fonc.2025.1709110 (PMC12827520; doi:10.3389/fonc.2025.1709110)
Supplement: Supplementary file 3 [file Table1.docx]

**Supplementary table1. Details of Gene Mutation Detection Results.**

| **Gene** | **Genotype** | **Chromosome** | **Position** | **Ref** | **Alt** | **Func.refGene** | **ExonicFunc.refGene** | **Change.refGene** |
| --- | --- | --- | --- | --- | --- | --- | --- | --- |
| JAK1 | heterozygous | 1 | 65307278 | T | C | exonic | nonframeshift insertion | NM_002227.4:exon18:c.A2410G:p.R804G |
| JAK1 | heterozygous | 1 | 65307282 | T | TTGTTTACAGAAAGAGAGA | exonic | nonframeshift insertion | NM_002227.4:exon18:c.2405_2406insTCTCTCTTTCTGTAAACA:p.K802delinsNLSFCKQ |
| PTPN6 | heterozygous | 12 | 7065710 | C | CTCTCGGGTGGTCATGA | exonic | frameshift insertion | NM_002831.6:exon9:c.1053_1054insTCTCGGGTGGTCATGA:p.T351fs |
| MTOR | heterozygous | 1 | 11307725 | A | ATCTCTGAT | exonic | frameshift insertion | NM_001386500.1:exon8:c.1181_1182insATCAGAGA:p.N394fs |
| TYK2 | heterozygous | 19 | 10476552 | A | ACTGCATCCC | exonic | nonframeshift insertion | NM_001385197.1:exon7:c.651_652insGGGATGCAG:p.S218delinsGMQS |
